# Supplementary material for: Meta-control of social learning strategies
Source: PLoS Comput Biol. 2022 Feb 28;18(2):e1009882. doi: 10.1371/journal.pcbi.1009882 (PMC8912904; doi:10.1371/journal.pcbi.1009882)
Supplement: S1 Text — (PDF) [file pcbi.1009882.s001.pdf]

# S1 Supporting information of the paper: Meta-control of social learning strategies[1]

## Contents

**S1.1 Population dynamics** 1

**S1.2 Learning from perfect and random models** 4

**S1.3 Sensitivity analysis: mutation rate** 6

**S1.4 Binary reward distributions** 6

**S1.5 Training environment** 7

**S1.6 Evolutionary optimization of the SL-GA** 7

**S1.7 Evolutionary optimization of the SL-NE** 8

**S1.8 Experiment design and results** 10

**S1.9 Evolution of meta-social learners: age distributions** 13

**S1.10 Evolution of meta-social learners: sensitivity analysis** 14

## S1.1 Population dynamics

We present the results of the mathematical model at the top row of Fig A and the evolutionary algorithm at the bottom row of Fig A. Notably, these approaches produce very similar results. The social learners using the success-based strategy show dominance over individual learners throughout the learning process as well as rapid adaptation after the reward reversal. On the other hand, the social learners using the conformist strategy show dominance only after the majority of the individuals learn to make optimum choices. When the latency is increased, the success-based strategy shows similarity to the results of the conformist strategy.

In highly uncertain environment, the success-based social learners lose their dominance in the population throughout the evolutionary processes (see Fig B). This indicates that the fitness of individual learners is higher than that of the success-based social learners. On the other hand, the behavior of conformist learners in uncertain environments is similar to that in static environments.

[1]Authors: Anil Yaman (a.yaman@vu.nl), Nicolas Bredeche, Onur Çaylak, Joel Z Leibo, Sang Wan Lee (sangwan@kaist.ac.kr)

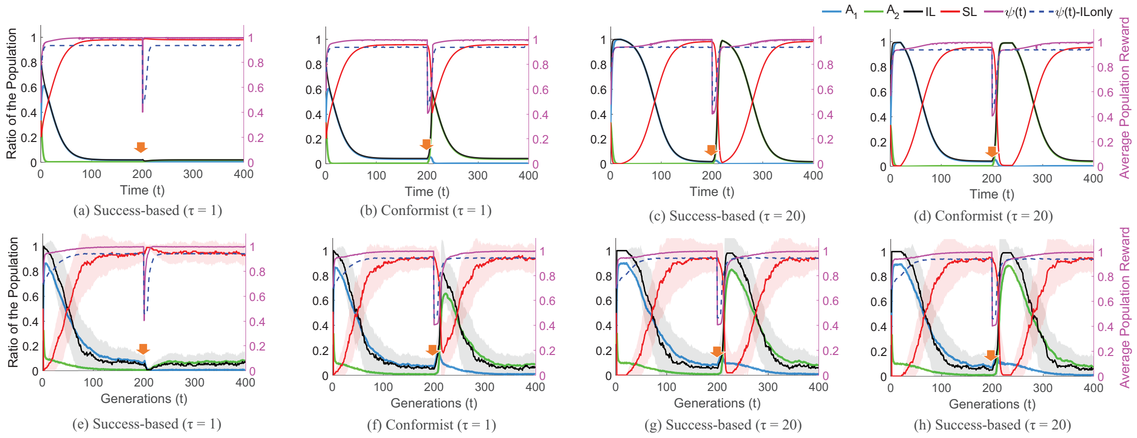

**Fig A. The population dynamics produced by the mathematical model ((a) through (d)) and evolutionary algorithm ((e) through (h)) on a 2-armed bandit (binary decision-making) task with reward distributions $R_1 \sim \mathcal{N}(1, 0.05)$ and $R_2 \sim \mathcal{N}(0.4, 0.05)$.** The first two columns show the results when the latency ($\tau$) for the social learners equals to 1, whereas, the figures in last two column show the results when it is set to 20. The $x$ and left $y$ axes show the ratio of individual and social learners in the population, and the right $y$ shows the average population reward (fitness) $\psi(t)$. $A_1(t)$ and $A_2(t)$ show the ratios of the individual learners that chose the first and second arms at $t$ respectively ($A_1(t) + A_2(t) = IL(t)$).

The population dynamics provided in Fig A and Fig B show the ratio of the strategies (individual versus specified social learning strategy) in the populations. In these processes, there is constant mutation (with certain rate) in each generation. Thus, we observe that the strategies converge on a certain ratio and dominate one another depending on the environment uncertainty. For instance, in environments with low uncertainty, success-based strategy becomes dominant throughout the evolutionary process (see Fig A (a)). However, when the uncertainty is increased, individual learning becomes dominant against success-based strategy (see Fig B (a)). Conformist strategy is the dominant strategy against individual learning in environments with low and high uncertainty (see Fig A (b) and Fig B (b)). After a reward reversal, an increase in the ratio of individual learners (and decrease in the ratio of conformist learners) can be observed until the majority of the population learns to perform new optimum behavior. Then, conformist strategy becomes the dominant strategy again. This is due to the fact that conformist strategy does not involve the learning cost and can perform well in environments with low and high uncertainty.

To summarize these results, and provide more clear visualization of stability and the basin of attraction of individual versus social learners starting from various initial conditions. Fig C provides the results in terms of the change in the ratio of the specified social learning strategy (versus individual learning strategy) in the populations. Each line shows the average of multiple runs of the evolutionary processes starting from a different initial conditions (number of individual versus social learning strategies in the population) in environments with low and high uncertainty. The ratios of

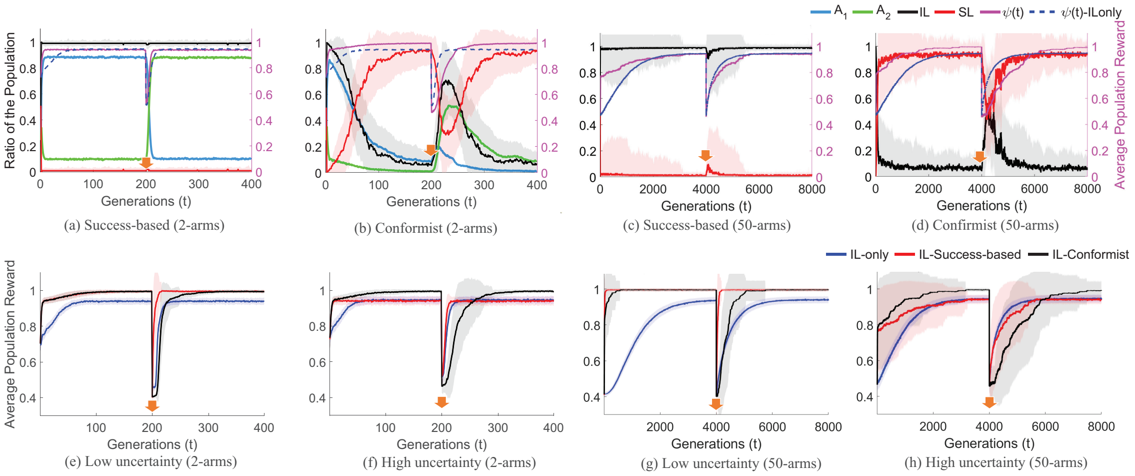


(a) Success-based (2-arms) (b) Conformist (2-arms) (c) Success-based (50-arms) (d) Confirmist (50-arms)

(e) Low uncertainty (2-arms) (f) High uncertainty (2-arms) (g) Low uncertainty (50-arms) (h) High uncertainty (50-arms)

**Fig B. (a) through (d) the population dynamics of the evolutionary processes in uncertain environments, and (e) through (h) the average population reward ($\psi(t)$) in populations consisting of only individual learners, individual learners with success-based social learners, and individual learners with conformist social learners in environments.** The uncertainty is introduced by increasing the standard deviation of the reward distribution of sub-optimum arm (optimum distribution: $R_1 \sim \mathcal{N}(1, 0.05)$, and sub-optimum distribution: $R_2 \sim \mathcal{N}(0.4, 0.5)$). Highlighted areas indicate the standard deviations of independent runs of the evolutionary algorithm. In all figures, orange arrows mark the reward reversal where the optimum and sub-optimum reward distributions are swapped. The visualization of the ratios of the individual arms in 50 armed case is omitted due to the large number of arms.

individual and social learning strategies sums up to 1 (thus, the individual learning strategy behaves inversely in terms of the change of their ratios in the populations relative to the social learners, $IL = 1 - SL$).

In the case of success-based strategy, success-based social learners become the dominant strategy in the population throughout the evolutionary process in an environment with low uncertainty. Even after a reward reversal, social learners quickly learn to choose the new optimum arm. In environments with high uncertainty on the other hand, success-based strategy cannot perform well, therefore individual learning becomes the dominant strategy throughout the evolutionary process.

In the case of conformist strategy on the other hand, when the environment is static (before and after a reward reversal), the ratio of strategies stabilize where non-dominant strategy (individual learning) fails to take over the population. However, after a reward reversal, the ratio of the conformist strategy decreases in the population until the optimum arm is learned by the majority of the individuals (due to the increase in the rate of individual learners). Then, individual learning becomes costly therefore conformist social learning strategy takes over the populations. Note that the change in the rate of the conformist strategy is similar in environments with high and low uncertainty.

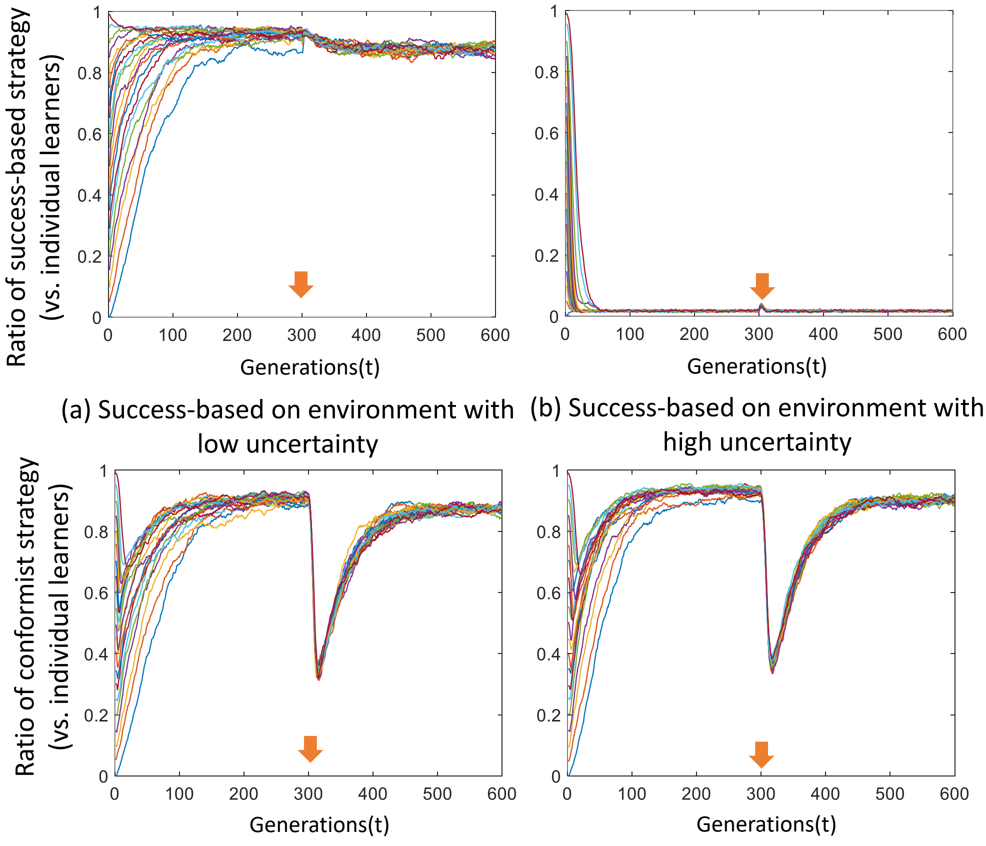


(a) Success-based on environment with low uncertainty (b) Success-based on environment with high uncertainty

(c) Conformist on environment with low uncertainty (d) Conformist on environment with high uncertainty

**Fig C. Stability and basin of attraction of the strategies (individual learning vs. a social learning strategy) during the evolutionary processes starting from the different initial conditions on environments with low and high uncertainty.** Reward reversal points are indicated by orange arrows on the x-axes. Each generation there is a constant mutation rate of 5e-3, therefore, dominant strategies do not reach to the ratio of 1.

## S1.2 Learning from perfect and random models

Fig D provides the results of three additional learning processes where social learners learn from a random individual, perfect model, or model that is 90% correct. The results show that, success-based and conformist strategies achieve similar performance as the perfect model in environment with low uncertainty, however, when there is high uncertainty in the environment, only conformist strategy achieves similar performance achieved by the perfect model. Learning from a model that is 90% correct achieves similar performance with individual learning only. This is due to the fact that individual learning has an exploration cost of 10% (exploration of other actions with 0.1 probability).

Learning from a random agent does not provide any improvement relative to the individual learning, and it performs worse when the environmental uncertainty is high. These results support our conclusions that the conformist strategy mitigates the effect of uncertainty in the environment and provides reliable performance independent of the environmental uncertainty.

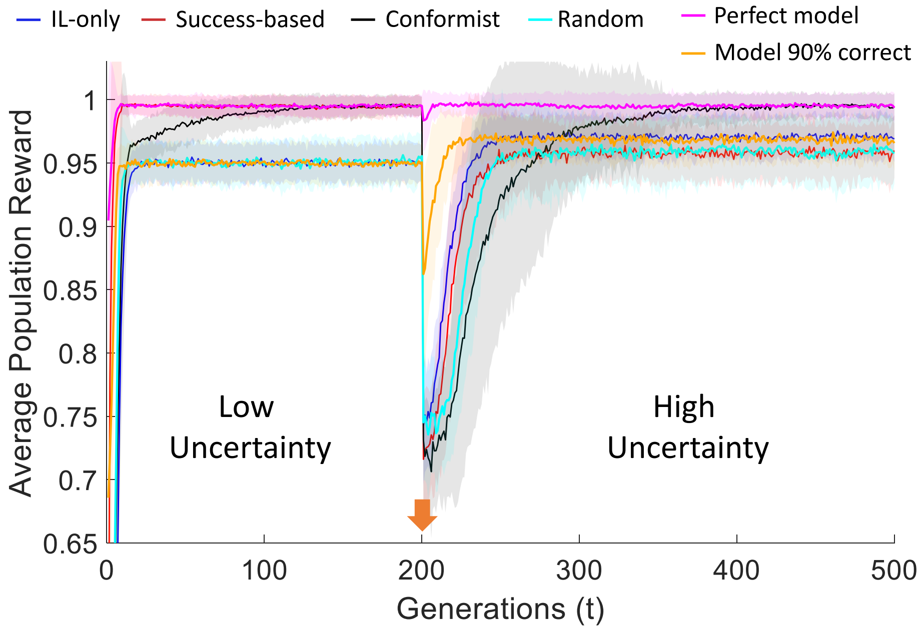


**Fig D. The comparison of success-based and conformist strategies to other three social learning approaches that does not require the knowledge of the most successful individual and the action of the majority.** In case of "Random" agents learn from a randomly selected agent in the population. "Perfect model" and "Model 90% correct" assume a model agent in the population that performs the correct behavior all the time, and 90% of the time respectively. In these two cases, the other agents in the population learn from the actions of these model agents. Reward reversal point is highlighted by the orange arrow.

## S1.3 Sensitivity analysis: mutation rate

The effect of mutation rate in the evolutionary analysis performed in Section 2.1 is illustrated in Fig E. In environments with low uncertainty, individual learning provides a lower bound for social learning, therefore, when the mutation rate is increased, the performance of social learners approaches the performance of individual learning. In environments with high uncertainty, conformist strategy performs similarly. However, in success-based strategy we observe the opposite effect in relative to the individual learning. In this case, individual learning provides an upper bound for success-based strategy and higher mutation rates cause divergence from the performance of individual learning. This is due to the fact that in environments with high uncertainty, success-based social learners perform worse than individual learners in the populations. This causes lower performance since higher mutation rates introduce more success-based social learners into the populations.

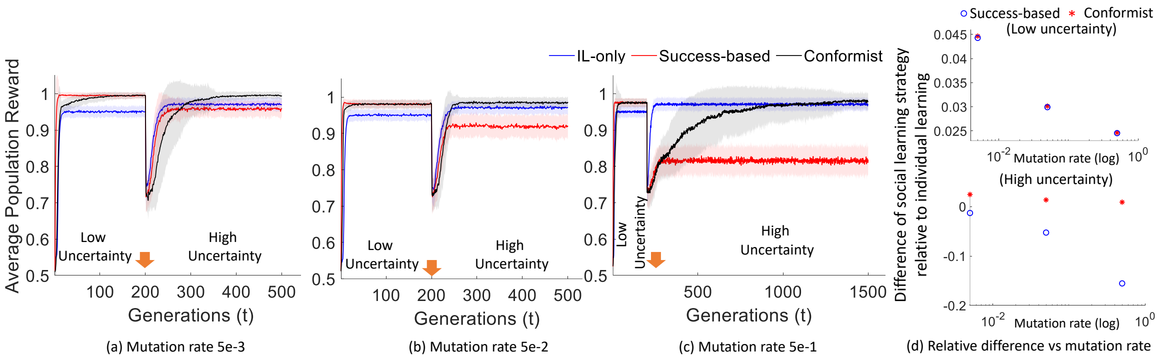


**Fig E. Higher mutation rates reduce the performance (in terms of average population reward) of the social learning strategies since higher mutation rates introduce more randomly mutated individuals that do not necessarily perform optimally (low uncertainty:**$\mu_1 = 1$, $\sigma_1 = 0.05$, $\mu_2 = 0.5$, $\sigma_2 = 0.05$; **high uncertainty:**$\mu_1 = 1$, $\sigma_1 = 0.05$, $\mu_2 = 0.7$, $\sigma_2 = 0.5$). (d) shows the difference of the social learning strategies relative to individual learning depending on mutation rate. While mutation rate increases, the performance of social learning strategies decreases and approaches to individual learning, however, in a high uncertainty environment individual learning performs better relative to success-based therefore the difference with individual learning increases in the negative direction.

## S1.4 Binary reward distributions

In case of binary reward distributions, the reward distributions can be modeled to provide binary rewards with certain probabilities. For example, if there are two arms A and B, with probabilities $\mu_1$ and $\mu_2$ of providing binary rewards, and there are $N$ and $M$ individual selecting these arms respectively, then success-based social learning selects one of these arms with the probabilities of $\frac{\mu_1 N}{(\mu_1 N+\mu_2 M)}$ and $\frac{\mu_2 M}{(\mu_1 N+\mu_2 M)}$ respectively. Assuming the optimum arm (that provides binary rewards more frequently, $\mu_1 > \mu_2$) is A, then, clearly, the lower $\mu_2$ is the higher the probability of copying

an individual that selects the optimum arm. On the other hand, conformist social learners can keep selecting the optimum arm independent of these probabilities as long as $N > M$ (that is when a larger number of individuals learn to select the optimum arm which is achieved by individual learning).

Fig F shows the simulation results demonstrating the performance of individual, conformist and success-based social learning strategies when the reward distributions of the arms are binary. Indeed, conformist strategy achieves a higher average population reward, and shows robust performance independent of $|\mu_1 - \mu_2|$.

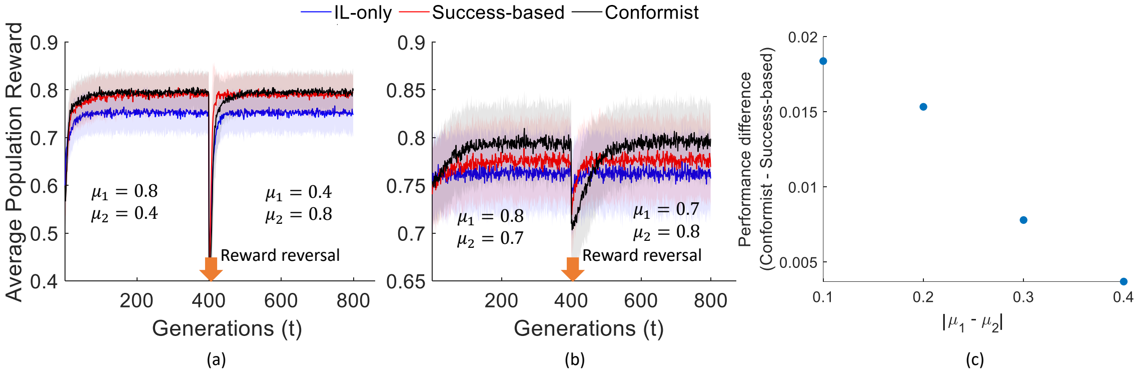


**Fig F. Conformist social learning performs better (in terms of average population reward) than success-based in case when the reward functions are binary.** The probabilities of providing binary rewards for each arm indicated with $\mu_1$ and $\mu_2$. Reward reversal points illustrated with orange arrows. Highlighted areas show one standard deviation from the mean. (c) shows that while $|\mu_1 - \mu_2|$ decreases, the performance difference between conformist and success-based strategies increases (all tested differences are statistically significant $p < 0.05$).

## S1.5 Training environment

Fig G shows the environment used for training phase of the algorithms. The trained algorithms then tested on separate environments and reported in the main text of the paper.

## S1.6 Evolutionary optimization of the SL-GA

In this section, we provide the results of the evolutionary processes of the GA used to optimize the decision policies in the SL-GA. We performed 10 independent GA runs on the environment shown in Figure Fig G. Fitness values are the median of the cumulative rewards of 112 processes.

The fitness trends during the GA processes, and the fitness versus standard deviations of the best solution (SL-GA policies) are shown in Fig H.

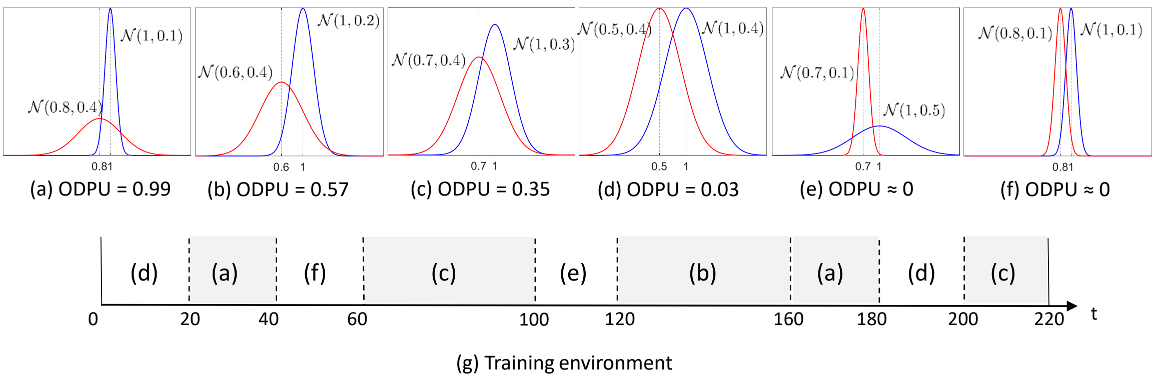


**Fig G. The environment used for training processes for algorithms: SL-GA and SL-NE.** (a) through (f) show arbitrarily defined reward distributions and their ODPUs for 2 arms. (g) shows the training environment generated by using the specified reward distributions for specified lengths of periods. The complete period consists of 220 time steps. Dashed vertical lines indicate the change of the reward distribution points. The periods with uncertainty (ODPU$\geq$ 0.1) are highlighted in gray.

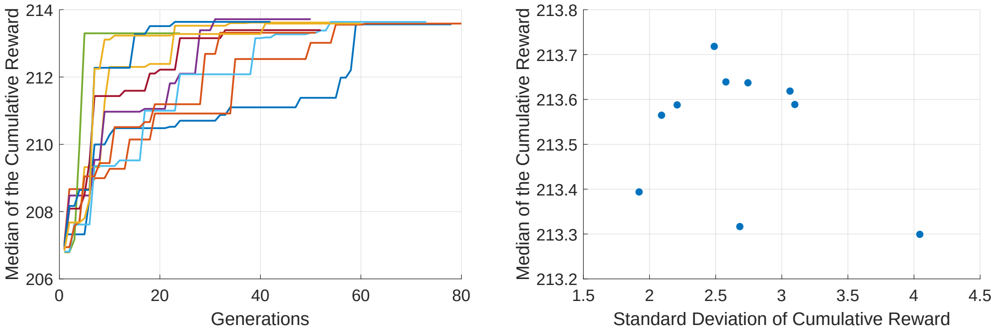


**(a)** Fitness trends **(b)** Fitness vs. standard deviation

**Fig H. (a) Fitness trend of 10 independent GA runs, and (b) fitness versus standard deviations of the best solution for each independent GA runs.**

## S1.7 Evolutionary optimization of the SL-NE

Fig I shows the evolutionary optimization processes of the ANN based policies using genetic algorithms and differential evolution. The evolutionary processes are terminated if there is no fitness improvement for 50 consecutive generations.

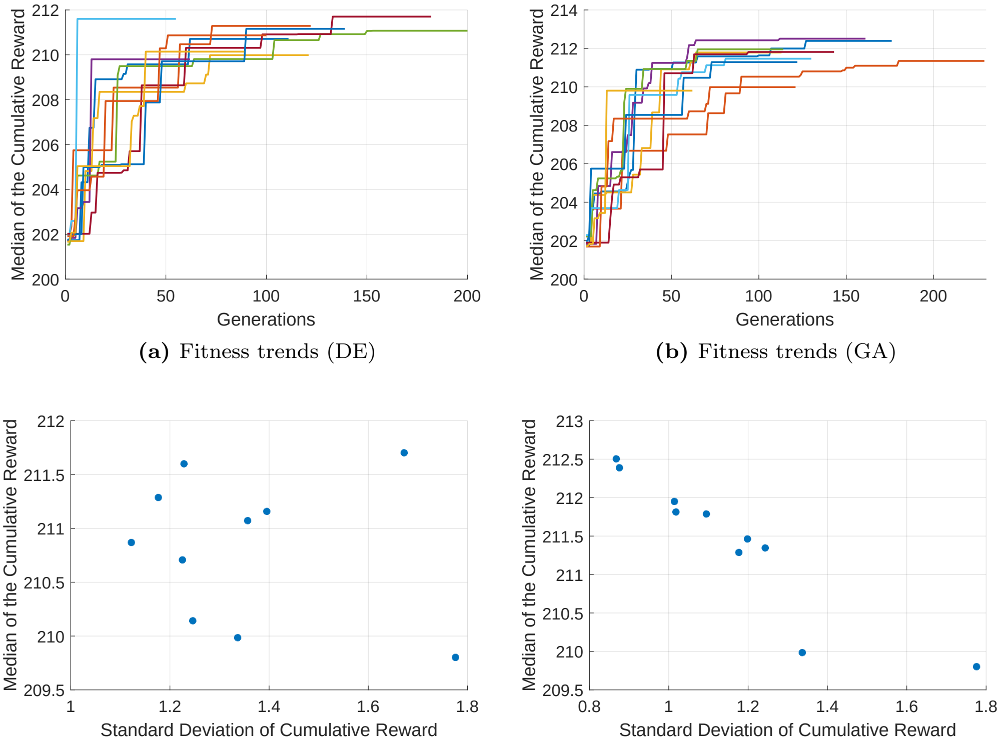


**(a)** Fitness trends (DE) **(b)** Fitness trends (GA)

**(c)** Fitness vs. standard deviation (DE) **(d)** Fitness vs. standard deviation (GA)

**Fig I. The evolutionary process of the ANNs using Neuroevolution approach.** (a) and (b) fitness trends of 10 independent DE and GA runs, and (c) and (d) fitness versus standard deviations of the best solution for each independent DE and GA runs.

## S1.8 Experiment design and results

We designed three sets of experiments with various volatility and uncertainty in terms of environment change and the overlap between the reward distributions. In **Experiment1**, we designed four environments as: stable low uncertainty, stable high uncertainty, volatile low uncertainty and volatile high uncertainty. In stable environments, the reward distributions were changed two, and in volatile environments five times. We defined six reward distributions (shown in Fig J) and assigned to a time period in the processes as illustrated in Fig L (a), (b), (c) and (g). Low and high uncertainty environments have low and high ODPUs respectively.

In **Experiment2** (random volatile environment), we defined random environments by selecting the number of environment change between $[10, 30]$ from uniform distribution, and assigned a reward distribution (from Fig J) to each period between environment change points randomly.

In **Experiment3** (gradual environment), we defined gradual environment change by defining the reward distributions based on sinusoidal functions as shown in Fig K.

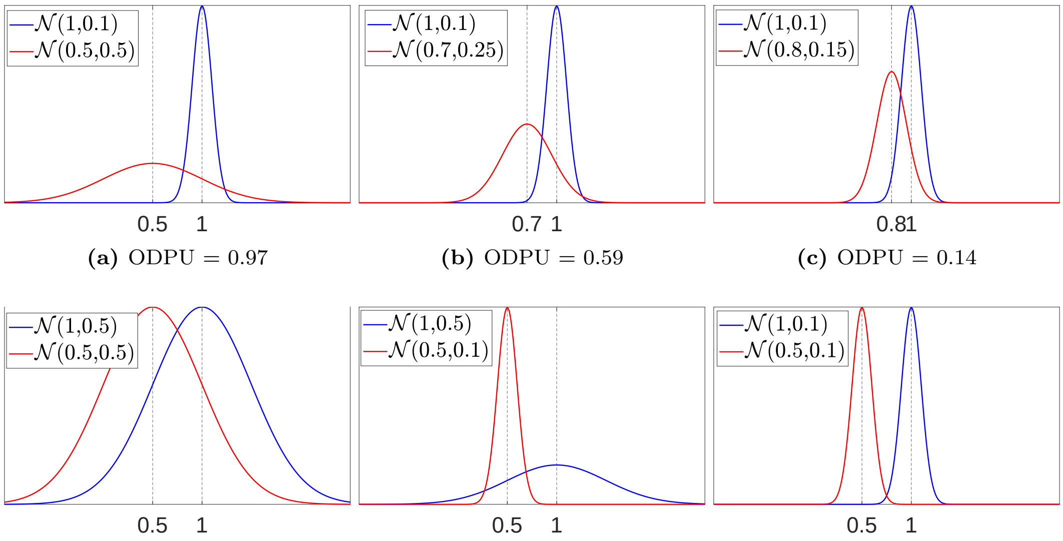


**(a)** ODPU $= 0.97$ **(b)** ODPU $= 0.59$ **(c)** ODPU $= 0.14$

**(d)** ODPU $= 0.10$ **(e)** ODPU $\simeq 0$ **(f)** ODPU $\simeq 0$

**Fig J. The reward distributions of the arms defined in each period of the environments shown on the $x$-axes of Fig L (a), (b), (c) and (g).**

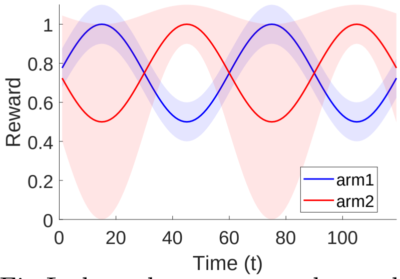


**Fig K. Reward distributions and their standard deviations (highlighted) in gradual environment.** We used two sinusoidal functions to model environment change. The standard deviation of the first arm kept constant while an additional sinusoidal function is used to model the change in the standard deviations of the second arm depending on time.

Fig L show the average and cumulative average population rewards obtained by the meta-social learning strategies in all experiments. Figures that show the performance versus exploration cost, and ranks of the strategies are given in Fig L.

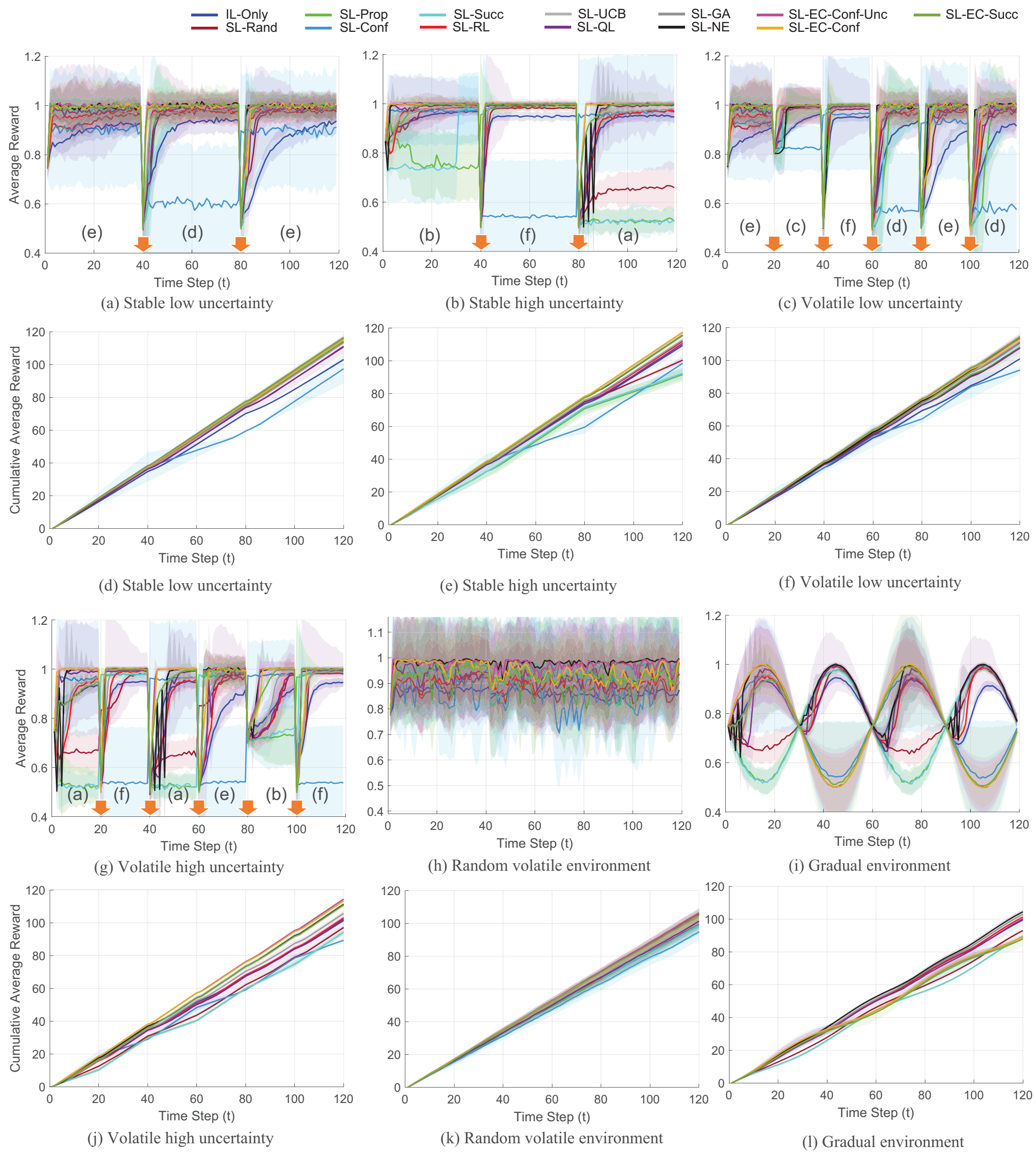


**Fig L. The average and cumulative average population rewards obtained by the meta-social learning strategies throughout the processes.**

The average results and standard deviations (highlighted) are based on 112 independent runs of each algorithm. Each orange arrow in $x$-axes indicates a reward reversal time point.

## S1.9 Evolution of meta-social learners: age distributions

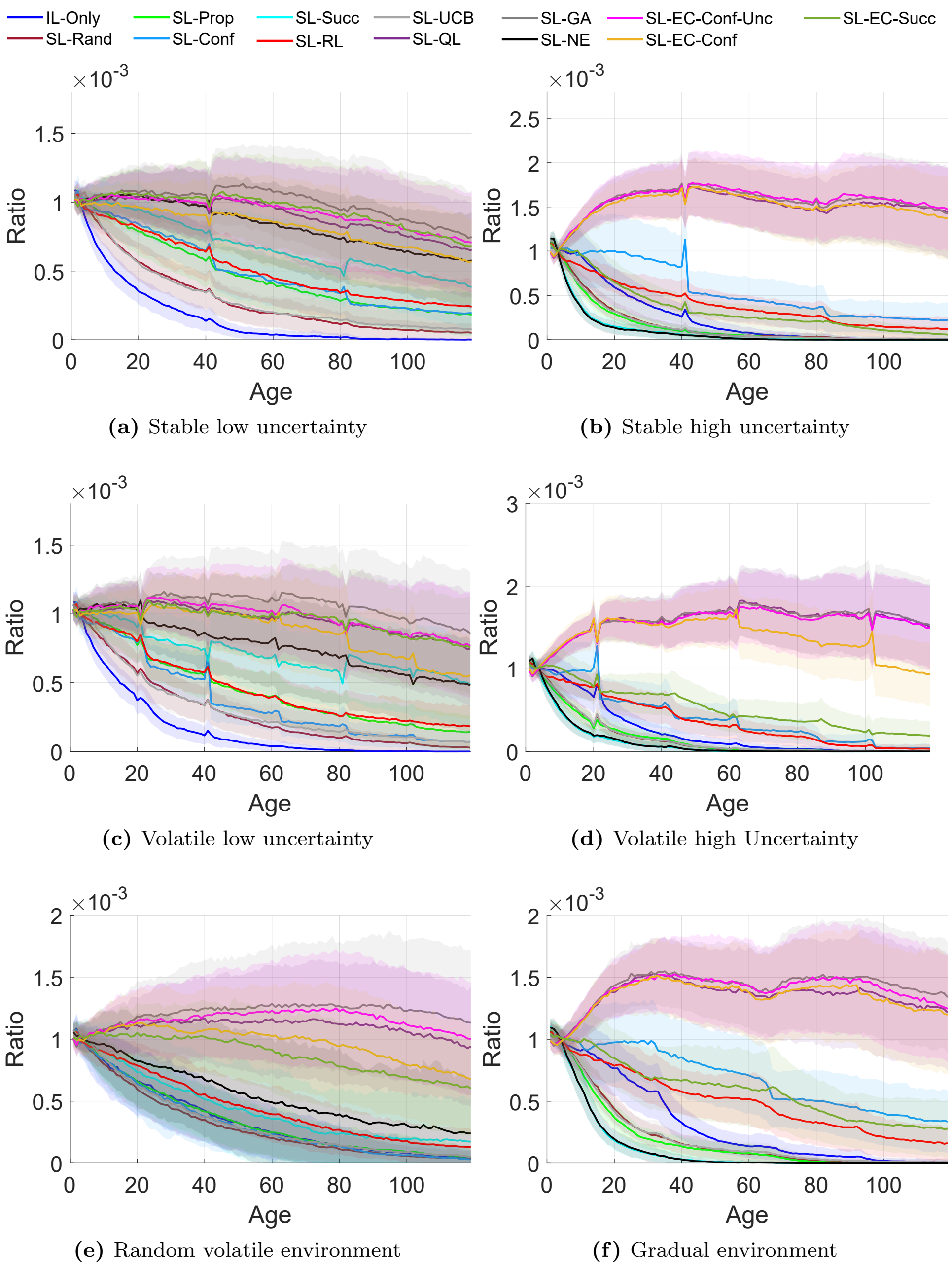


**(a)** Stable low uncertainty

**(b)** Stable high uncertainty

**(c)** Volatile low uncertainty

**(d)** Volatile high Uncertainty

**(e)** Random volatile environment

**(f)** Gradual environment

**Fig M. The age distributions of the meta-social strategies throughout the evolutionary processes.** The age distributions of the dominant strategies are higher relative to others.

## S1.10 Evolution of meta-social learners: sensitivity analysis

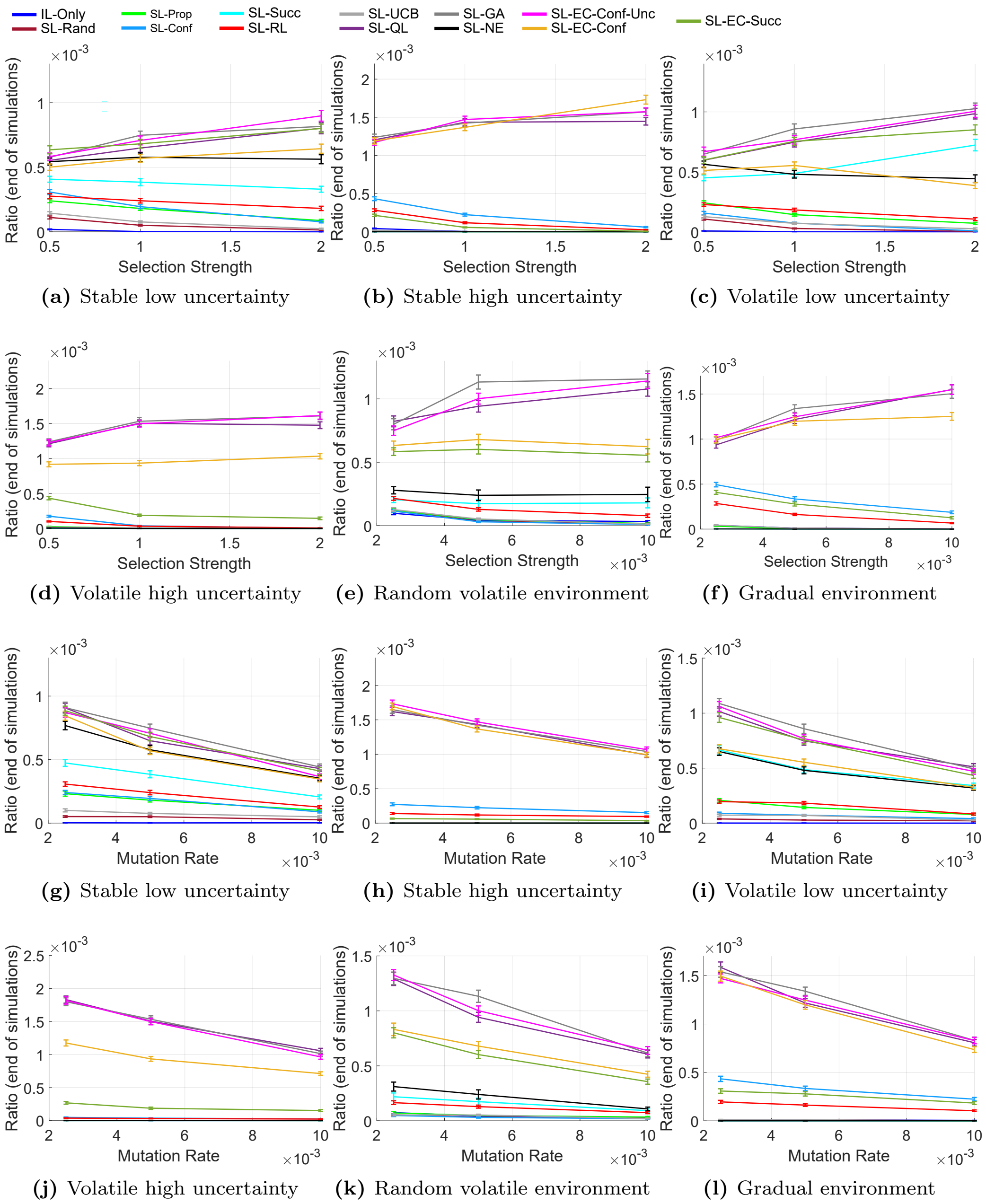


**Fig N. The effect of selection strength and mutation rate to the domination of the successful strategies.** While the selection strength increases, domination of successful strategies increases; however, while the mutation rate increases, domination of successful strategies decreases.
