# Supplementary figures and images for: Meta-control of social learning strategies
Source: PLoS Comput Biol. 2022 Feb 28;18(2):e1009882. doi: 10.1371/journal.pcbi.1009882 (PMC8912904; doi:10.1371/journal.pcbi.1009882)

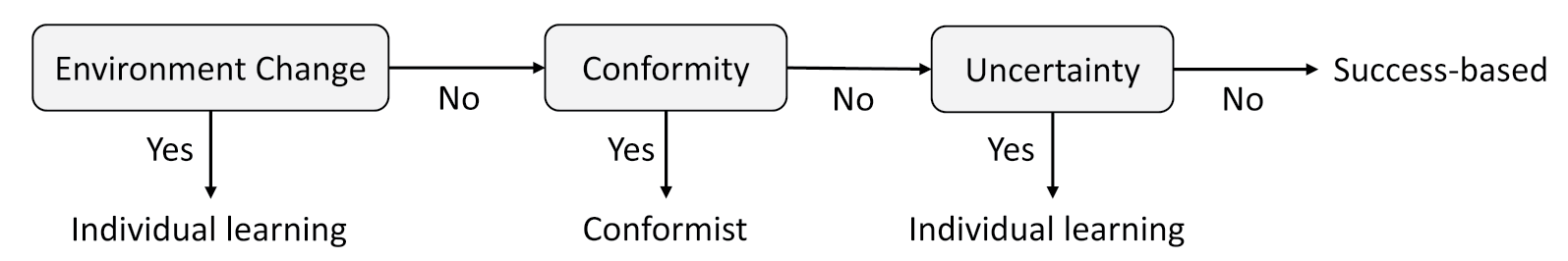

Supplement: S1 Diagram — “Yes” and “No” indicate 1 and 0 states of the environmental variables shown in Table 2. Thresholds of the evolved rule for uncertainty and environment change is thu = 0.05 and thec = 0.15. (TIF) [file pcbi.1009882.s002.tif]

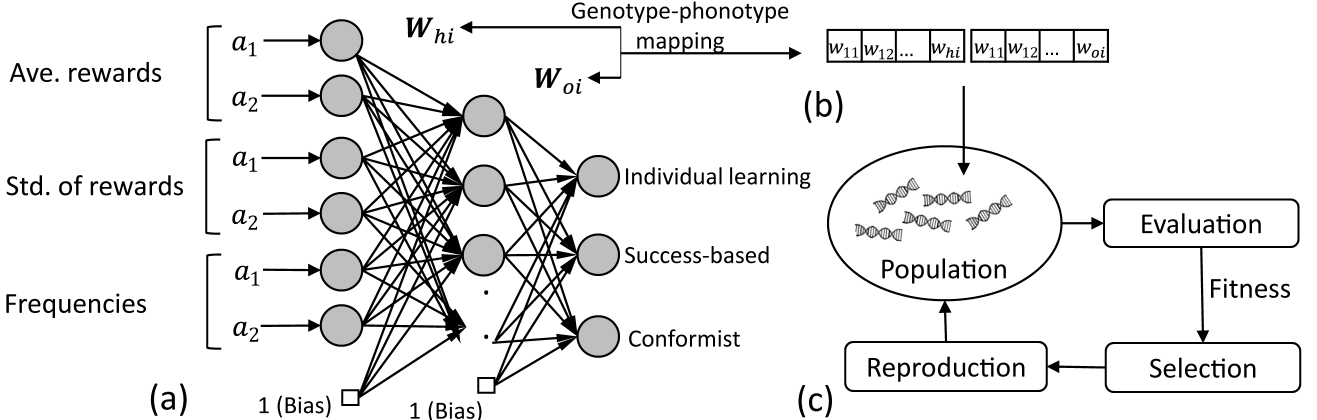

Supplement: S1 Fig — (a) Feed-forward artificial neural network topology with one hidden layer can take the average, standard deviations and frequencies of two actions a1 and a2 and decides to perform individual learning, success-based or conformist social learning strategies. (b) The weights of the networks between input and hidden layers (Whi), and hidden and output layers (Woi) are directly mapped to the genotype of the individuals and represented as real valued vectors. (c) Evolutionary algorithms are used to optimize the genotype of the individuals. (TIF) [file pcbi.1009882.s003.tif]
