# Supplementary material for: Meta-control of social learning strategies
Source: PLoS Comput Biol. 2022 Feb 28;18(2):e1009882. doi: 10.1371/journal.pcbi.1009882 (PMC8912904; doi:10.1371/journal.pcbi.1009882)
Supplement: Attachment — Submitted filename: Response Letter.pdf [file pcbi.1009882.s004.pdf]

Response letter: Meta-control of social learning strategies

We thank all the reviewers for their constructive comments and questions. Below, we provide our answers, colored as blue, after each comment. In addition to the changes regarding the comments, we made the code for the experiments publicly available at:
https://github.com/anilyaman/Metacontrol-of-social-learning-strategies

Reviewer #1: Yaman and colleagues examined the performance of the social learning strategies in volatile and uncertain environments. Comparison between success-based and conformity-based social learning strategies based on the simulation data demonstrated that success-based social learning works better in the low-uncertainty environment. In contrast, the conformity-based strategy works better in high-uncertainty environments. Furthermore, they characterised an arbitration mechanism (i.e., meta-control) of the learning strategies that resolves environmental uncertainty with minimal exploration cost. I believe this study addressed the critical issue and would potentially provide a unified framework of social learning that spans evolutionary biology, psychology, neuroscience, and machine learning.

One of my major concerns is the conceptual validity of success-based social learning. The success-based strategy requires an agent to keep track of the others' actions as well as their reward outcomes. If each agent has the cognitive capacity of doing so, s/he can use another form of social learning: that is, learning from others' outcomes (i.e., observational learning). Indeed, recent studies in social neuroscience have suggested that humans combine learning from others' actions and that from others' rewards. It would be great if the authors could test for the case.

Conceptual validity of success-based learning: Thank you for your comment and suggestion. We added the paragraph below in introduction (lines 109-118) to address this point.

"Individual learning was modeled as a value-based approach known as the ϵ-greedy algorithm where the agents aim to figure out the arm that provides the highest average reward by trial-and-error (Sutton and Barto, 2018) (see Section 4.2). In the case of social learning, success-based and conformist social learning strategies were modeled based on copying the actions of the most successful agent and majority of the agents respectively (see Section 4.3). This assumes that the success-based strategy has access to the knowledge of the action of the most successful individual (in terms of the rewards received), and the conformist strategy assumes the knowledge of the action that is performed by the majority of the individuals in the population. This is in line with the hypothesis that suggests that humans, in particular, possess domain specific cognitive capabilities for social learning to allow them to assess the knowledge and performance of others (Heyes, 2016; Kendal et al., 2018; Olsson, 2020; Whiten et al. 2021)."

Heyes, C. (2016). Who knows? Metacognitive social learning strategies. *Trends in cognitive sciences*, *20*(3), 204-213.

Kendal, R. L., Boogert, N. J., Rendell, L., Laland, K. N., Webster, M., & Jones, P. L. (2018). Social learning strategies: Bridge-building between fields. *Trends in cognitive sciences*, *22*(7), 651-665.

Olsson, A., Knapska, E., & Lindström, B. (2020). The neural and computational systems of social learning. *Nature Reviews Neuroscience*, *21*(4), 197-212.

Whiten, A., Biro, D., Bredeche, N., Garland, E., & Kirby, S. (2021). The emergence of collective knowledge and cumulative culture in animals, humans and machines. Philosophical Transactions of the Royal Society B: Biological Sciences.

Observational learning: Thank you for your comment regarding observational learning. We agree that investigating observational learning would be an interesting and relevant extension to this work. Therefore, we added two paragraphs below to Discussion, and in addition to that, we included three social learning experiments that do not necessitate success-based and conformist strategies (i.e. keeping track of successful agent, and the action of the majority):

Below, we provide the paragraphs added to Discussion:

"The success-based and conformist social learning strategies modeled in this work assume that the individuals have access to the knowledge of the action of the most successful individual, and the action performed by the majority respectively. One interesting approach is to model the learning mechanisms to learn based on the choices of other agents (e.g. observational learning (Charpentier et al., 2020, Colette et al. 2017)). Since the prior works were concerned with exploring key variables, albeit in limited settings, to determine an individual to copy or infer underlying goals, our framework could be extended by incorporating these features.

Our neural network based meta-control strategy (SL-NE) can be viewed as a form of observational learning. In this case, our network takes the frequencies of actions and rewards received by agents in the population as inputs and decides which action to take. More elaborate version of this model can identify which individual to copy by looking at the actions and rewards of all individuals. However, testing these models should outweigh various computational issues. For example, the addition of such features essentially increases the size of the input space for the same task, and may not provide further understanding of the optimality of the social learning strategies depending on the environment uncertainty as studied in this work. We acknowledge that understanding neural mechanisms to identify individuals to copy their behavior or infer their goals based on population dynamics is a very interesting research direction. Future works could directly benefit from our meta-control social learning frameworks. For example, variants of the SL-NE can be used to explore new neural hypotheses in more general settings."

Charpentier, C. J., Iigaya, K., & O'Doherty, J. P. (2020). A Neuro-computational account of arbitration between imitation and emulation during human observational learning. Neuron 106 (4).

Collette, S., Pauli, W. M., Bossaerts, P., & Doherty, J. O. (2017). Neural computations underlying inverse reinforcement learning in the human brain, 1-20

Furthermore, below we provide the results of the additional experiments and include them in Section S1.2 S1 Text. These additional results provide further support for your analysis, and are in line with our conclusions.

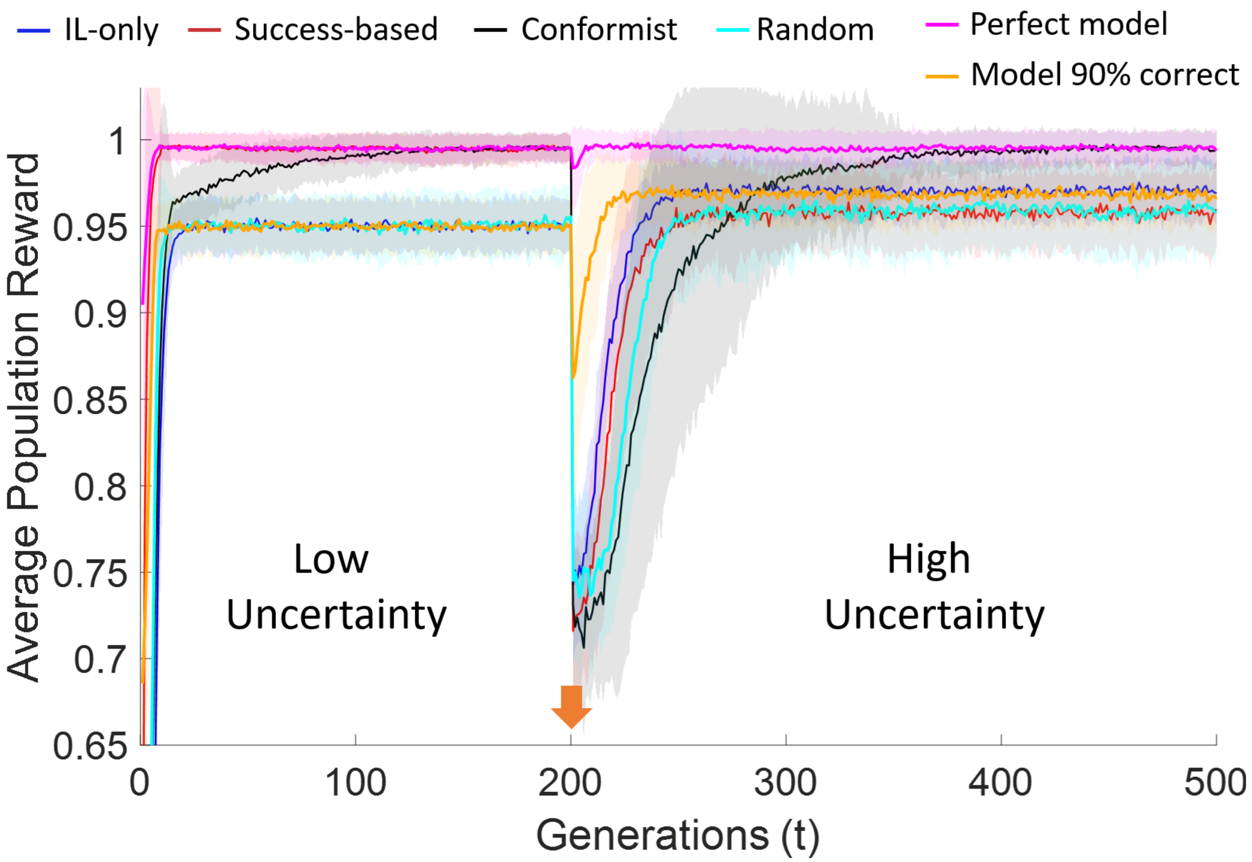

Figure 1. The comparison of success-based and conformist strategies to other three social learning approaches that does not require the knowledge of the most successful individual and the action of the majority. In the case of “Random” agents learn from a randomly selected agent in the population. “Perfect model” and “Model 90% correct” assume a model agent in the population that performs the correct behavior all the time, and 90% of the time respectively. In these two cases, the other agents in the population learn from the actions of these model agents. Reward reversal point is highlighted by the orange arrow.

The results show that success-based and conformist strategies achieve similar performance as the perfect model in an environment with low uncertainty, however, when there is high uncertainty in the environment, only conformist strategy achieves similar performance achieved by the perfect model. Learning from a model that is 90% correct achieves similar performance with individual learning only. This is due to the fact that individual learning has an exploration cost of 10% (exploration of other actions with 0.1 probability). Learning from a random agent does not provide any improvement relative to the individual learning, and it performs worse when the environmental uncertainty is high. These results support our conclusions that the conformist strategy mitigates the effect of uncertainty in the environment and provides reliable performance independent of the environmental uncertainty.

I am also concerned with the robustness of their conclusions. The simulations apparently have a lot of parameters to be determined in an arbitrary way (e.g., |mu_1 – mu_2|, sigma, th_ec, tc_u). In other words, the overall conclusions of the study may be changed with different sets of

parameter values. For instance, the mutation rate is known to have the potential to dramatically change the evolutionary phenomena from the stational to oscillatory and chaotic dynamics (Nowak & Sigmund, PNAS 1993). The authors could perform exhaustive sensitivity analyses in order to check the robustness of their findings with respect to the choice of parameter values. Furthermore, I am wondering what happens in the case of binary reward outcome (reward or no-reward), as previous studies have mainly focused on binary reward.

Test environments: Thanks a lot for insightful comments. We believe that the environment cases included in our analysis involved a wide range of scenarios for supporting our conclusions. For instance, we defined six cases with various degrees of uncertainty and volatility (detailed in Section S1.8 in S1 Text). Four of these cases have been defined based on the combinations of high and low uncertainty (various degrees of overlap between two reward distributions), and number of reward reversals (2 and 5) (Experiment 1). Additionally, in one case we select the number of reward reversals (between [10, 30]) and the reward distributions randomly (Experiment 2), and finally, in another case, the parameters of the reward distributions change gradually modeled by sinusoidal functions (Experiment 3).

Sensitivity analysis: To highlight this point we added the following text to the end of Section 2.1:

“The evolutionary parameters, in this case mutation rate, is expected to have an effect in the evolutionary dynamics (Nowak & Sigmund, PNAS 1993). We performed sensitivity analysis with various mutation rates and showed the results in Section S1.3 in S1 Text. We observe the following effect in line with our conclusions: the higher the mutation rate, the higher the rate in which randomly mutated individuals (as individual or social learners) are introduced into the populations. Since randomly mutated individuals would not perform optimally, higher mutation rates cause a reduction in the performance of the populations with social learners.”

And, we added the text and figure below in Section S1.3 in S1 Text that supports our conclusions:

The effect of mutation rate in the evolutionary analysis performed in Sections 2.1 is illustrated in Figure 2 (below). In environments with low uncertainty, individual learning provides a performance lower bound for social learning, therefore, when the mutation rate is increased, the performance of social learners approaches the performance of individual learning.
In environments with high uncertainty, conformist strategy performs similarly. However, in success-based strategy we observe the opposite effect in relative to the individual learning. In this case, individual learning provides a performance upper bound for success-based strategy and an increase in mutation rates decreases the performance of individual learning. This is due to the fact that in highly uncertain environments, success-based social learners perform worse than individual learners in the populations. This causes lower performance since higher mutation rates introduce more success-based social learners into the populations.

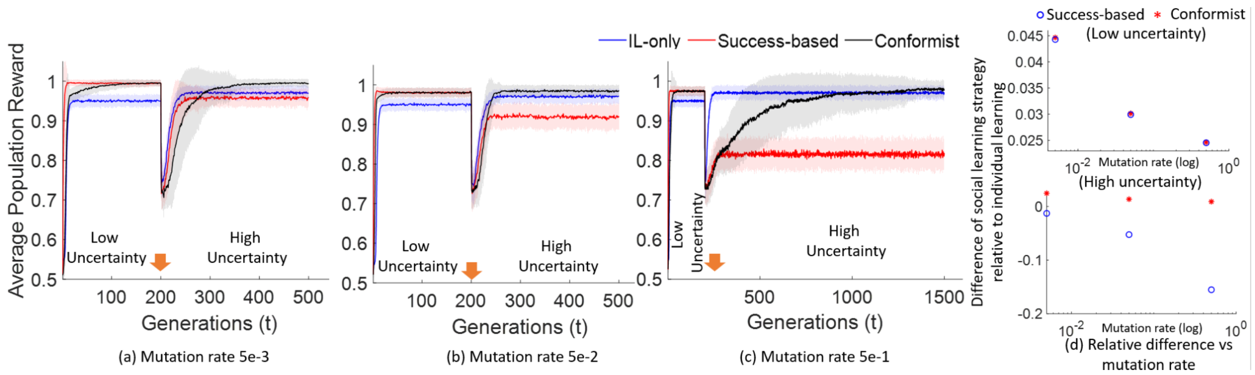

Figure 2. Higher mutation rates reduce the performance (in terms of average population reward) of the social learning strategies since higher mutation rates introduce more randomly mutated individuals that do not necessarily perform optimally (low uncertainty: $\mu_1 = 1,\ \sigma_1 = 0.05,\ \mu_2 = 0.5,\ \sigma_2 = 0.05$;high uncertainty:$\mu_1 = 1,\ \sigma_1 = 0.05,\ \mu_2 = 0.7,\ \sigma_2 = 0.5$). (d) shows the difference of the social learning strategies relative to individual learning depending on mutation rate. While mutation rate increases, the performance of social learning strategies decreases and approaches to individual learning, however, in a high uncertainty environment individual learning performs better relative to success-based therefore the difference with individual learning increases in the negative direction.

In terms of the evolutionary parameters (selection pressure and mutation) for the analysis performed on the evolution of meta-social learning strategies in Section 2.5, we provide the sensitivity analysis in Section S1.10 in S1 Text. Based on this analysis, (as stated in the last paragraph of the Results section) we found that, while the selection strength increases, dominance of successful strategies increases; however, while the mutation rate increases, dominance of successful strategies decreases. This is due to the fact that, when the mutation rate is high, the probability of randomly mutating a dominant strategy increases, causing their life expectancy to decrease.

Binary reward distributions: Thank you for the constructive suggestion. We added the following text to the end of Section 2.1.

“We performed an additional analysis with binary reward functions, the case often implicated in many reinforcement learning studies. This scenario would not involve uncertainty in the same way studied in this work (i.e. given by the overlap between the Gaussian reward distributions). However, the reward distributions can be modeled to provide binary rewards (i.e. without the loss of generality, the arms can either provide 1 or 0, or two other arbitrary reward values) with certain probabilities μ_1 and μ_2, and in this case, smaller differences between these probabilities cause higher uncertainty in identifying successful individuals in the population to copy. Simulation results for this case are presented in Section S1.4 in S1 Text. Our simulations showed that decrease in the difference between μ_1 and $\mu_2|$ causes the performance of the success-based strategy to decrease. On the other hand, conformist strategy achieves a higher average population reward relative to success-based strategy, and shows

robust performance independent of $|\mu_1-\mu_2|$. We note that the results of this additional analysis are aligned fully with our results. Therefore, it can provide additional evidence and further support for our conclusions regarding the effect of the environment uncertainty to success-based strategy.”

And, to support our conclusions, we added the text and figure below to Section S1.4 in S1 Text:

In binary reward scenarios, the reward distributions can be modeled to provide binary rewards with certain probabilities. For example, if there are two arms A and B, with probabilities μ_1 and μ_2 of providing binary rewards, and there are N and M individual selecting these arms respectively, then success-based social learning selects one of these arms with the probabilities of $\frac{\mu_1 N}{(\mu_1 N + \mu_2 M)}$ and $\frac{\mu_2 M}{(\mu_1 N + \mu_2 M)}$ respectively. Assuming the optimum arm (that provides binary rewards more frequently, $\mu_1 > \mu_2$) is A, then, clearly, the lower μ_2 is the higher the probability of copying an individual that selects the optimum arm. On the other hand, conformist social learners can keep choosing the optimum arm independent of these probabilities as long as $N > M$ (that is when a larger number of individuals learn to select the optimum arm, which is achieved by individual learning).

Figure 3 (given below) shows the simulation results demonstrating the performance of individual, conformist and success-based social learning strategies when the reward distributions are binary. Indeed, conformist strategy achieves higher average population reward, and shows robust performance independent of $|\mu_1-\mu_2|$.

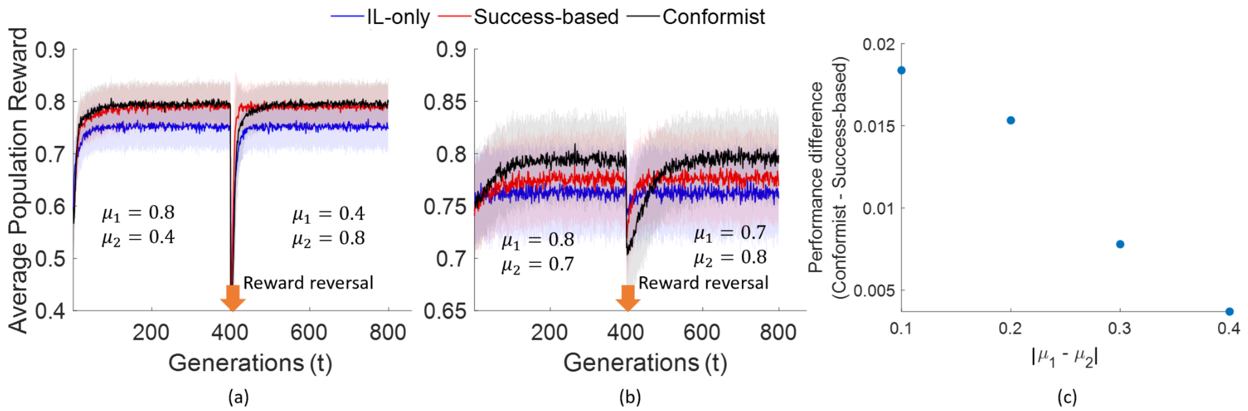

Figure 3. (a) and (b) show that conformist social learning outperforms (in terms of average population reward) than success-based in case when the reward functions are binary. The probabilities of providing binary rewards for each arm indicated with $\mu_1$and μ_2. Reward reversal points illustrated with orange arrows. Highlighted areas show one standard deviation from the mean.
(c) shows that while $|\mu_1 - \mu_2|$ decreases, the performance difference between conformist and success-based strategies increases (all tested differences are statistically significant $p < 0.05$).

To explore the evolutionary dynamics, researchers often examine the evolutionary stability (i.e., whether the population dominated by the strategy can resist the invasion of a small number of

individuals who employ another strategy), as well as the basin of attraction (i.e., how much the strategy dominates the population depending on the different initial distributions of the strategies). The authors could address those points.

Stability and basin of attraction: thank you for your comments. Indeed, we agree that it is informative to discuss these points in the text. We added the text below (highlighted in Section 2.1) to refer to the population dynamics, stability and basin of attraction analysis provided in Section S1.1 in S1 Textl.

"For detailed analysis of the population dynamics, and the stability analysis and basin of attraction, see Section S1.1 in S1 Text. In addition, in Section S1.2 in S1 Text, we compare the performance of success-based and conformist strategies with two social learning cases where individuals copy a perfect and random model. These cases do not require the knowledge of the successful individual and the decision of the majority."

In Section S1.1 in S1 Text, we added the following text and figure provided below:

The population dynamics provided in S1A Fig and S1B Fig show the ratio of the strategies (individual versus specified social learning strategy) in the populations. In these processes, there is constant mutation (with certain rate) in each generation. Thus, we observe that the strategies converge on a certain ratio and dominate one another depending on the environment uncertainty. For instance, in environments with low uncertainty, success-based strategy becomes dominant throughout the evolutionary process (see S1A Fig (a)). However, when the uncertainty is increased, individual learning becomes dominant against success-based strategy (see S1B Fig (a)). Conformist strategy is the dominant strategy against individual learning in environments with low and high uncertainty (see S1A Fig (b) and S1B Fig (b)). After a reward reversal, an increase in the ratio of individual learners (and decrease in the ratio of conformist learners) can be observed until the majority of the population learns to perform new optimum behavior. Then, conformist strategy becomes the dominant strategy again. This is due to the fact that conformist strategy does not involve the learning cost and can perform well in environments with low and high uncertainty.

To summarize these figures, and provide more clear visualization of stability and the basin of attraction of individual versus social learners starting from various initial conditions. Figure 4 (given below) provides the results in terms of the change in the ratio of the specified social learning strategy (versus individual learning strategy) in the populations. Each line shows the average of multiple runs of the evolutionary processes starting from a different initial conditions (number of individual versus social learning strategies in the population) in environments with low and high uncertainty. The ratios of individual and social learning strategies sums up to 1 (thus, the individual learning strategy behaves inversely in terms of the change of their ratios in the populations relative to the social learners, IL = 1 - SL).

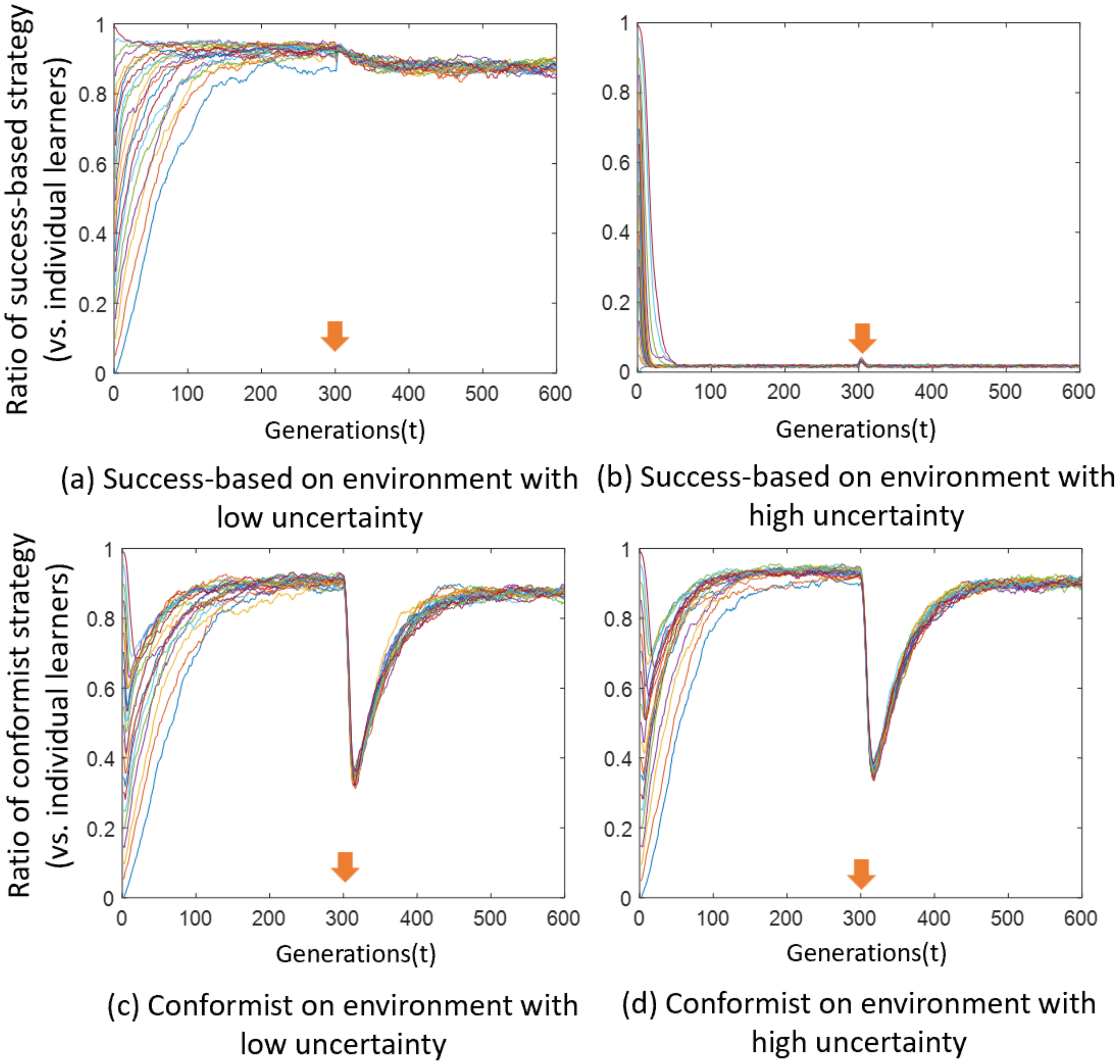

(a) Success-based on environment with low uncertainty (b) Success-based on environment with high uncertainty

(c) Conformist on environment with low uncertainty (d) Conformist on environment with high uncertainty

Figure 4. Stability and basin of attraction of the strategies (individual learning vs. a social learning strategy) during the evolutionary processes starting from the different initial conditions on environments with low and high uncertainty. Reward reversal points are indicated by orange arrows on the x-axes. Each generation there is a constant mutation rate of 5e-3, therefore, dominant strategies do not reach to the ratio of 1.

In the case of success-based strategy, success-based social learners become the dominant strategy in the population throughout the evolutionary process in an environment with low uncertainty. Even after a reward reversal, social learners quickly learn to choose the new optimum arm. In environments with high uncertainty on the other hand, success-based strategy cannot perform well, therefore individual learning becomes the dominant strategy throughout the evolutionary process.

In the case of conformist strategy on the other hand, when the environment is static (before and after a reward reversal), the ratio of strategies stabilize where non-dominant strategy (individual

learning) fails to take over the population. However, after a reward reversal, the ratio of the conformist strategy decreases in the population until the optimum arm is learned by the majority of the individuals (due to the increase in the rate of individual learners). Then, individual learning becomes costly therefore conformist social learning strategy takes over the populations. Note that the change in the rate of the conformist strategy is similar in environments with high and low uncertainty.

We discuss the domination (in terms of their proportion in the populations) of the meta-social learning approaches during the evolutionary processes in Section 2.5. Figure 5 (in the paper) shows the ratios of the meta-social learning approaches in the populations. Here, we can observe the domination of successful strategies through the evolutionary processes. These figures and analysis provide the stability analysis of these strategies in changing environmental conditions within a wide range of uncertainty levels.

In economics, social learning has been studied in the context of 'information cascade', which demonstrates an adverse effect of social learning. Furthermore, the authors pointed out the possibility that exploration is costly. One fascinating question is who incurs the cost and who free-rides others' exploration (e.g., see Bolton and Harris, Econometrica 1999). I am wondering if the present study has implications for those issues.

Thank you for your comment and the reference. Yes, this is a very interesting question. Two paragraphs in introduction (from lines 69 to 87) of our original manuscript discuss the cost and benefits of individual and social learning strategies depending on the environmental context. Although "free-riding" was not specifically mentioned, we discussed that individual learning incurs the cost while social learning takes the advantage of the exploration performed by others (thus, free-rides). Of course, if there is too much free-riding (that is if a large number of individuals is performing social learning), then there will be no exploration and the population will get stuck on a sub-optimum behavior especially if the environment is changing.

Highlighted in introduction we rephrase the paragraph below (see line 79 in the text) and included the reference to explicitly address this point:

"In group-living animals in nature, social learning has evolved to take advantage of the exploration performed by others via copying their behavior (i.e. ``free-riding'' the exploration performed by the others without incurring the cost (Bolton and Harris, 1999), thereby reducing the cost of learning. Therefore, it does not involve these costs related to individual learning [26, 32, 62, 5]. On the other hand, if there are too many individuals that take advantage of social learning then it may not be possible to explore other behaviors to find the optimum behavior especially in changing environments."

The key concept of the paper is the optimum distribution prediction uncertainty (ODPU). I believe it would be helpful to explain the concept in a concise way in the main text (e.g., in the Introduction or Section 2.2). I was a little bit confused with the concepts of uncertainty, risk (sigma) and volatility. For example, for me, it was difficult to understand the statement like "we

hypothesised that the high uncertainty in the environment would make it hard to identify successful individuals (lines 138-139)" without the clear definition of uncertainty.

Thank you for your comments. We made several changes highlighted in the text to clarify concepts. In case of the environment uncertainty, these changes are in lines: 105, 174. Section 2.2 aims to provide a more precise definition of environment uncertainty measured by the ODPU by also providing mathematical definition in Methods, Section 4.4.

In case of stable and volatile environments, we added a brief explanation in line 315.

The legends of Figures 2, 4 and 5 could be more informative. I would like the authors to provide more detailed information in the legends, so that the naïve readers understand, for example, how to read out Fig 2a and the critical difference (CD) diagrams and so on.

Thank you for your comment. Highlighted in the captions, we have extended the descriptions of Figures 2, 4 and 5.

In some parts, the authors reported p-values of the statistical test. Does it make sense to perform the statistical test on the simulated data (where the sample size is not meaningful)?

Since it is a common practice to use statistical tests to show the significance of the difference between two datasets statistically, we used these tests to show the significance of the difference between the results of multiple runs (e.g. 112 runs in total) of different simulations and algorithms. Furthermore, to provide additional indicators, we also show the standard deviations of these results highlighted on each figure or shown in form of error bars. To address this point, we added the following sentence to footnote 5:

“Furthermore, we provide the standard deviations of the results of multiple runs highlighted on each figure or shown in form of error bars”

Reviewer #2: In this work, the authors propose an approach to social learning, which they name meta-social learning.

They justify their approach on studies on brain skills on learning strategies and cognitive-behavioral science. The authors deal with a trade-off between environmental uncertainty and performance-cost rate by a model that explores individual learning and two different social learning strategies, namely success-based and conformist. These strategies are well-known in the field of social learning.

To do this, the authors use a common set-up, the multi-armed bandit, implementing two different dynamics: replicators and agent-based.

Via numerical simulations, they find that in uncertain environments, the conformist strategy performs better than the success-based strategy, In general, none of the three strategies achieve an optimal policy for lifetime learning. Therefore, the authors propose a mixed strategy, the so-called meta-social learning strategy, to fit in an environment characterized by both volatility and uncertainty. The proposed meta-social learning strategy uses estimated uncertainty to arbitrate between the three pure strategies (individual learning, success-based and conformist).

They successfully tested their model with a large set of different well-known algorithms.

I like the paper as it is and recommend its publication.

A couple of minor remarks (optional):

I find confusing the Panel a of Figure 1. I am not sure how to read it (at least, I find much easier the main text to understand the model).

Thank you for your comment. We have updated Figure 1 and separated two populations as: individual and conformist learners, and individual and success-based learners to illustrate the copying process of the conformist and success-based social learners. We hope the figure could convey the message more clearly now.

The authors may consider taking a look to this paper:

Cardoso, F.M. et al. Dynamics of heuristics selection for cooperative behaviour. New Journal of Physics, 22(12), p.123037. (2020)

Thank you for pointing out the interesting reference. We included the reference into the discussion section. Indeed, it may be very interesting to study further social learning strategies in cooperative task settings.

Reviewer #3: This paper is about discussing whether to learn individually or to imitate other strategies in a group, in the uncertainty of the environment.

First of all, at the beginning, I wanted it to say that individual learning here means e-greedy RL, see 4.2, should be clearly stated. Also what we are really doing in social learning is found in 4.3, should be mentioned. I couldn't figure that out and had a hard time reading the analogy the whole time.

Thank you for your comments and suggestions. Highlighted, we have added clarification in the caption of Figure 1 and text in line 95.

On top of that, the results are not very surprising. What happens when the degree of uncertainty is varied continuously? The definition of uncertainty is given too easy or to formalistic. Agents should resolve the uncertainty or to infer the behind. The present approach is too much top down (or GOFAI) and I didn't learn anything from here.

Thank you for your comment. In fact, we did perform an analysis on different cases with various difficulties including the cases where the degree of uncertainty varied randomly with high

random volatility as well as the case where it changed continuously and gradually (detailed in Section S1.8 in S1 Text).

Social learning has evolved to reduce the cost of learning, however, it raises a fundamental challenge that is to identify which individuals to copy. Although success-based and conformist strategies are two most widely studied social learning strategies, the tradeoff of these strategies, especially in uncertain and volatile environments, is largely unaddressed. Surely, seeking a suitable compromise between individual and social learning strategies is crucial for optimal learning as a population.

To follow up on this research agenda, one of our main motivations in this work is to identify the key environmental variables that can play a crucial role in the performance difference between success-based and conformist strategies. We show that the environment uncertainty (formalized by the ODPU) is such a variable. Therefore, we hypothesized and tested the premise that ODPU can be an effective predictor in switching between strategies to perform an optimal learning. Therefore, we believe that this "top-down" analysis and results not only make a contribution with this knowledge but also can inform the design processes of more efficient and effective multi-agent learning systems.

Indeed, it is an interesting research direction to design "bottom-up" learning processes that can emerge to perform individual and social learning. Therefore, we have also tested bottom-up approaches in the paper such as the ones that are discovered by genetic algorithms and neural networks (i.e. SL-NE, SL-GA, SL-RL) and online learning approaches based on reinforcement learning. Especially in the case of the SL-NE, the decision mechanisms are found to switch between the strategies based without the input of the ODPU and compared with the "top-down" approach. We believe that there is a great potential in applying these approaches to other machine learning paradigms.

To summarize, the main contributions of this work are:

Uncertainty-invariance in social learning. Investigation of the susceptibility of social learning is environmental uncertainty. We show that, surprisingly, environmental uncertainty is one key factor that causes decrease in the performance of the success-based strategy, whereas the conformist strategy can effectively mitigate this adverse effect. This finding can have an important impact on multi-agent learning models that rely only on a success-based measure..

Performance-exploration tradeoff in the social context. Our analysis and the identification of key parameters that can play role in this tradeoff can provide a basis for resolving the performance-exploration tradeoff in the social context, and lead to more robust (that exhibit good performance in many different environments) decision-mechanisms.

Meta-social learning hypothesis. Accordingly, we hypothesized that meta-control of individual and social learning strategies, referred to as meta-social learning, serves as an effective and sample-efficient alternative to knowledge acquisition. The proposed meta-social learning scheme aims to find a near-optimal compromise between individual, success-based, and conformist strategies depending on the uncertainty of the environment. Our simulations showed that the proposed model can improve learning performance significantly while reducing the exploration cost.
